# Intrauterine exposure to preeclampsia does not impair vascular health in children

**DOI:** 10.3389/fpubh.2022.1071304

**Published:** 2022-12-22

**Authors:** Benjamin J. Varley, Amanda Henry, Lynne Roberts, Gregory Davis, Michael R. Skilton, Maria E. Craig, Megan L. Gow

**Affiliations:** ^1^The University of Sydney, Children's Hospital Westmead Clinical School, Sydney, NSW, Australia; ^2^Department of Paediatrics and Child Health, St George Hospital, Kogarah, NSW, Australia; ^3^Discipline of Paediatrics and Child Health, School of Clinical Medicine, UNSW Medicine and Health, University of New South Wales, Sydney, NSW, Australia; ^4^Discipline of Women's Health, School of Clinical Medicine, UNSW Medicine and Health, University of New South Wales, Sydney, NSW, Australia; ^5^Department of Women's Health, St George Hospital, Kogarah, NSW, Australia; ^6^The George Institute for Global Health, Sydney, NSW, Australia; ^7^School of Clinical Medicine, UNSW Medicine and Health, University of New South Wales, Sydney, NSW, Australia; ^8^Boden Initiative, Charles Perkins Centre, University of Sydney, Sydney, NSW, Australia; ^9^Sydney Medical School, University of Sydney, Sydney, NSW, Australia; ^10^Sydney Institute for Women, Children and their Families, Sydney Local Health District, Sydney, NSW, Australia

**Keywords:** preeclampsia, aortic intima-media thickness, pulse wave velocity, children, early arterial injury

## Abstract

**Background and objectives:**

Preeclampsia is a serious multisystem blood pressure disorder during pregnancy that is associated with increased long-term risk of cardiovascular disease to the mother and offspring. We investigated the vascular health of children exposed to intrauterine preeclampsia.

**Materials and methods:**

This was a cross-sectional study of offspring in a prospective cohort of women with complications during pregnancy. Children aged between 2 and 5 years [median age 4.7 (2.8, 5.1) years] exposed to intrauterine preeclampsia (*n* = 26) or normotensive controls (*n* = 34), were recruited between July 2020 and April 2021. Vascular health was assessed by measuring aortic intima-media thickness and pulse wave velocity. Univariate generalized linear regression models were used to explore associations between vascular measurements and explanatory variables.

**Results:**

Children exposed to preeclampsia had a lower body mass index at assessment (15.5 vs. 16.2 kg/m^2^, *p* = 0.04), birth weight (2.90 vs. 3.34 kg, *p* = 0.004), gestational age at birth (37.5 vs. 39.4 weeks, *p* < 0.001) and higher frequency of preterm birth (27% vs. 6%, *p* = 0.02). There were no differences in vascular health between children exposed to preeclampsia vs. controls (mean aortic intima-media thickness 0.575 mm vs. 0.563 mm, *p* = 0.51, pulse wave velocity 4.09 vs. 4.18 m/s, *p* = 0.54) and there were no significant associations in univariate analyses.

**Conclusions:**

There were no major adverse differences in vascular health which contrasts with existing studies. This suggests exposure to intrauterine preeclampsia may result in a less severe cardiovascular phenotype in young children. While reassuring, longitudinal studies are required to determine if and when exposure to intrauterine preeclampsia affects vascular health in children.

## Introduction

Preeclampsia is a multisystem disorder in pregnancy, characterized by pregnancy hypertension after 20 weeks' gestation and maternal and/or fetal organ dysfunction. Preeclampsia affects 3–5% of all pregnancies and is associated with increased maternal and infant mortality as well as adverse pregnancy outcomes including preterm birth and fetal growth restriction (1, 2). There is an increased long-term risk of cardiovascular disease (CVD) in these mothers as a result of preeclampsia (1), and there is growing evidence of increased long-term cardiovascular risk in children exposed to intrauterine preeclampsia (3, 4). Historically, research investigating the effects of preeclampsia exposure on children has primarily focused on identifying traditional risk factors such as increased body mass index (BMI) and blood pressure (BP).

Atherosclerosis and arteriosclerosis are key pathophysiological processes that result in the structural and degenerative changes of large arteries which underlie CVD. Measurements of intima-media thickness and pulse wave velocity (PWV) can assess such arterial wall changes and are recognized as acceptable, non-invasive measurements of early arterial injury in children (5–7), and can be used to predict potential risk of future CVD (8–10). Measurement of carotid intima-media thickness is widely used in adults for future CVD risk assessment (11). However, abdominal aorta intima-media thickness (aIMT) may be a more appropriate measure in children (6, 12, 13), as it is more sensitive to childhood risk factors including familial hypercholesterolemia, type 1 diabetes (13), and adverse outcomes during pregnancy such as fetal growth restriction (14, 15) and large for gestational age (16, 17). Preliminary evidence has shown greater aIMT in neonates exposed to preeclampsia compared to those unexposed (18–20), whereas effects on PWV during childhood are conflicting (21, 22).

The purpose of this study was to investigate whether vascular health in children aged 2–5 years of age is affected by exposure to intrauterine preeclampsia. The primary hypothesis was that children exposed to preeclampsia have signs of early arterial injury including increased aIMT and PWV, compared to the children of uncomplicated pregnancies.

## Materials and methods

This was a cross-sectional sub-study of offspring from participants in the Postpartum Physiology, Psychology and Pediatric follow up study (P4 study), a prospective, observational study of postpartum women with either normal BP or preeclampsia in their preceding pregnancy, conducted at St George Hospital, Sydney, Australia. A study protocol for the P4 cohort has been published (23).

Women were eligible for the P4 study if they gave birth to a singleton live baby within the previous 6 months and had a good understanding of written and spoken English. Women were excluded if they had chronic hypertension, diabetes, renal or other serious disease prior to pregnancy, were pregnant again at the time of first (6 months postpartum) assessment, or if their baby was born with a congenital anomaly.

The recruited cohort consisted of 90 women who had preeclampsia during pregnancy and 402 control women who had a normotensive pregnancy. Preeclampsia was defined as persistent *de novo* hypertension (systolic BP ≥ 140 mmHg and/or diastolic BP ≥ 90 mmHg) that developed at or after 20 weeks' gestation accompanied with one or more of the following new-onset conditions: proteinuria, other maternal organ dysfunction including liver or kidney involvement, neurological complications, low platelets or uteroplacental dysfunction, according to the guidelines of the International Society for the Study of Hypertension in Pregnancy (24, 25). Preeclampsia with severe hypertension was defined as systolic BP ≥ 160 mmHg and/or diastolic BP ≥ 110 mmHg (25) and onset was considered early if occurring before 34 weeks gestation (26).

All children born to mothers in the P4 cohort study were eligible for recruitment into this cross-sectional sub-study. Children between 2 and 5 years of age were recruited between July 2020 to April 2021. Children were excluded from analysis if neither aIMT nor PWV could be obtained. Previous study data examining aortic-IMT in growth-restricted neonates vs. controls [MD (SD), 0.07 (0.08)] (27) indicated a minimum required sample size of 22 children exposed to preeclampsia and 22 normotensive controls to detect a significant difference between groups (power = 0.8, α = 0.5). This sub-study was approved by the South Eastern Sydney Local Health District Human Research Ethics Committee (2019/ETH11984).

### Pediatric assessments

Height was measured using a stadiometer and weight with electronic scales. Height, weight and BMI z-scores were calculated according to WHO Child Growth Standards (28). Gestational age at birth, birth weight and length were collected from the mother's maternity medical records. Z-scores for birth weight and length according to gestational age were calculated for preterm (<37 weeks) and term infants using the INTERGROWTH-21st (29) and WHO Child Growth Standards, respectively (28). Small for gestational age was defined as a birth weight z-score below −1.28 for gestational age and sex, corresponding to the 10th percentile.

Vascular structure was assessed by aIMT which was measured using a GE Voluson S6 system with a linear array transducer (GE 9L-RS) and a frequency of 3–10 MHz, as previously described (13). High-resolution images of the far wall of a non-branched, 1 cm longitudinal segment of the abdominal aorta near the aortic bifurcation were captured. Gain was adjusted to optimize image quality. A minimum of two loops were captured for blinded offline analysis. Mean and maximum aIMT were calculated with validated edge detection software during end-diastole (Carotid Analyzer, Medical Imaging Applications LLC; Coralville, IA). AIMT was defined as the distance from the edge of the lumen intima to the media-adventitia border of the far wall. Aortic diameter was defined as the media-adventitia borders between near and far walls and was captured during end diastole. All scans and analyses were performed by one technician (BJV) after training from an experienced technician and followed a standardized protocol. AIMT scans were excluded if they lacked sufficient quality for analysis, including too much movement, poor image quality or presence of sonographic artifacts.

Vascular function was assessed by measuring PWV and central BP using the SphygmoCor XCEL (AtCor Medical, West Ryde, NSW, Australia) as described (30). Simultaneous recordings of pulse waveforms were obtained by placing an applanation tonometer on the right common carotid, whereas the femoral waveform was measured by volume displacement produced by a cuff placed around the upper thigh. The arterial path was measured directly using non-stretchable tape between the right common carotid and femoral arteries multiplied by 0.8. PWV was automatically calculated by the XCEL software system (Version 1.3) by dividing the arterial path distance by the transit time between the pulse waveforms. A standard brachial cuff was used to capture brachial and diastolic pressures and provide central BP. An average of three measurements was used for each assessment. PWV measurements were excluded if they did not meet quality control of the Sphygmocor device (31). Under optimal conditions, children rested for a minimum of 10 mins in a supine position before vascular measurements.

### Maternal assessments

Pregnancy data including antihypertensive medication, pregnancy outcome, dating scan, weight and BMI were obtained from the mother's maternity medical records. Demographic data including age, ethnicity, highest education level, previous diabetes and smoking status, alcohol intake, exercise and drug use were obtained from a questionnaire that the mother completed 6 months after birth.

### Data analysis

Descriptive statistical analyses are reported as mean ± SD or median (IQR). The primary outcome measures were aIMT and PWV. Secondary outcome measurements included aortic lumen diameter and central BP. Between group differences for continuous outcome measures were assessed using independent sample *t*-tests (normal distribution) or Mann-Whitney *U* Tests (non-normal distribution) and for categorical outcomes using Chi-square or Fisher's exact tests. Univariate generalized linear models were used to compare means, with adjustment for age and gender, and in a separate model, BMI and birthweight. Non-parametric variables were log transformed for univariate linear models.

Univariate generalized linear regression models were also used to explore the association between outcome measures and explanatory variables, including gestational age at birth, birth weight, birthweight Z-score, birth length, birth length z-score, gender, height, height Z-score, weight, weight Z-score, BMI, BMI Z-score, preterm, small for gestational age, maternal age, maternal dating scan BMI, maternal dating scan weight, maternal smoking ever, maternal moderate exercise at 6 months, maternal alcoholic drinks at 6 months, parity, antihypertensive medication in pregnancy, months breastfed, drug use ever, preeclampsia severity of hypertension, preeclampsia onset, highest maternal BP during pregnancy and preeclampsia exposure. Sub-group analysis of aIMT and PWV by preeclampsia severity of hypertension, onset, and gestational age were also performed. All statistical analyses were performed using SPSS (version 26; SPSS Inc., Chicago, IL, USA).

## Results

### Participant characteristics

**Figure 1:**
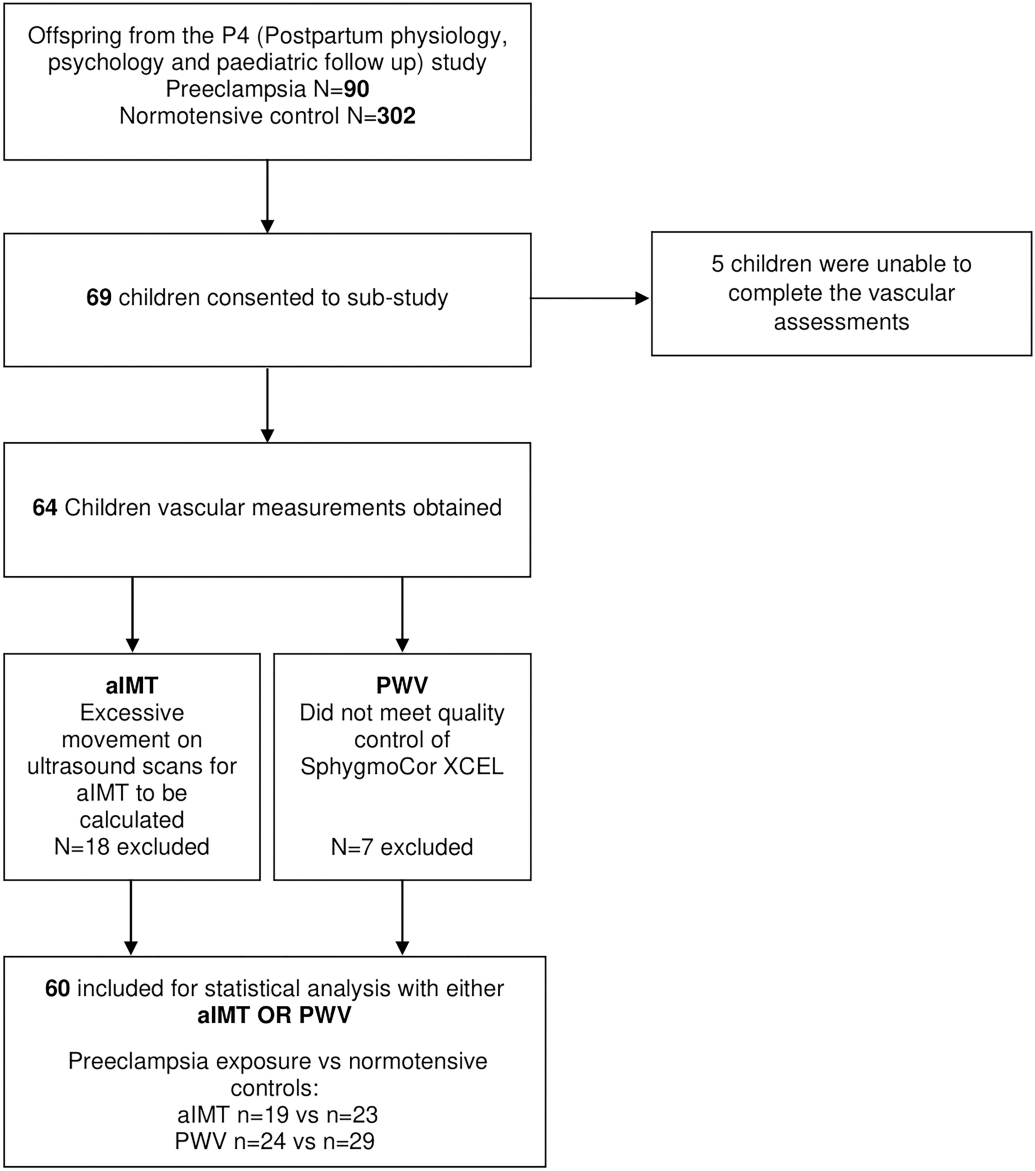
Participant recruitment and analysis flow diagram.

Sixty-nine children from the P4 study were consented to participate in this sub-study and underwent assessment. Five participants were excluded as they were unable to complete the vascular assessments at the study visit and a further four were excluded in quality control because neither aIMT nor PWV could be obtained (Figure 1). A total of 26 children exposed to intrauterine preeclampsia and 34 controls were included in the final analysis.

**Figure 2:**
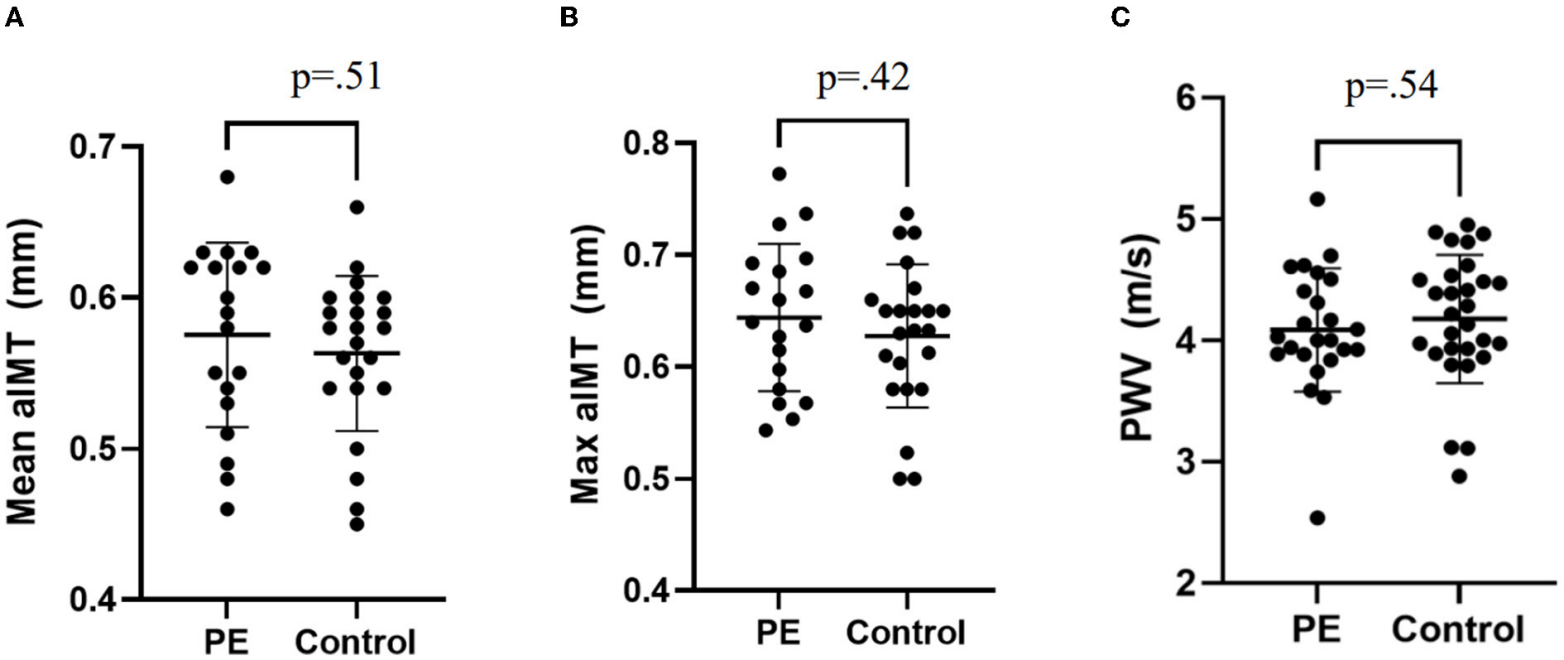
Individual patient data for vascular measures. **(A)** Mean aIMT, **(B)** Maximum aIMT, and **(C)** Pulse wave velocity. Plots presented as mean ± SD. PE, Preeclampsia.

The characteristics of participants are shown in Table 1. Children exposed to preeclampsia had a lower BMI at time of assessment (15.5 kg/m^2^ vs. 16.2 kg/m^2^), birth weight (2.90 kg vs. 3.34 kg), gestational age at birth (37.5 weeks vs. 39.4 weeks) and higher frequency of preterm birth (27% vs. 6%). There were no other differences between groups. Vascular measurements are presented in Table 2 and individual participant data are presented in Figure 2. Central systolic BP was higher in children exposed to preeclampsia when adjusted by BMI and birthweight. There were no differences in aIMT or PWV between groups.

**Table 1:** Offspring characteristics stratified by exposure to preeclampsia vs. normotensive pregnancy.

	**Preeclampsia *n* = 26**	**Control *n* = 34**	***p value***
Male	15 (58)	19 (56)	0.89
Age at assessment (years)	4.3 (3.2, 5.1)	5.0 (2.2, 5.3)	0.92
Height (cm)	107 (97.3, 112)	107 (89.4, 111)	0.46
Height Z-score	0.46 (−0.54, 0.87)	−0.18 (−0.71, 0.43)	0.15
Weight (kg)	17.2 (15.2, 19.4)	18.2 (13.4, 19.9)	0.99
Weight Z-score^*^	0.21 ± 1.00	0.30 ± 0.79	0.69
BMI (kg/m^2^)	15.5 ± 1.2	16.2 ± 1.2	**0.04**
BMI Z-score	0.05 ± 0.83	0.47 ± 0.80	0.05
Birth weight (kg)	2.90 ± 0.66	3.34 ± 0.46	**0.004**
Birth weight Z-score^*^	−0.33 ± 0.83	−0.07 ± 0.99	0.28
Birth length (cm)	48.5 ± 3.7	50.0 ± 2.4	0.06
Birth length Z-score^*^	−0.20 ± 1.01	−0.13 ± 1.12	0.81
Gestational age (weeks)	37.5 ± 2.3	39.4 ± 1.4	**<0.001**
Preterm birth	7 (27)	2 (6)	**0.02**
Months breastfed	11.1 ± 8.3	12.7 ± 6.8	0.41

Data presented as n (%), mean ± SD or median (IQR).

* Corrected for gestational age.

BMI, Body mass index.

The bold values indicate the statistical significance (*p* < 0.05).

**Table 2:** All vascular outcomes stratified by exposure to preeclampsia vs. normotensive pregnancy.

	**Preeclampsia**	**Control**	***p***	**Adjusted *p* value^a^**	**Adjusted *p* value^b^**
**aIMT**	*N* = 19	*N* = 23			
Mean aortic intima-media thickness (mm)	0.575 ± 0.06	0.563 ± 0.05	0.51	0.48	0.49
Max aortic intima-media thickness (mm)	0.644 ± 0.07	0.628 ± 0.06	0.42	0.45	0.62
Aortic lumen diameter (mm)	8.04 (7.62, 8.60)	7.55 (7.01, 8.25)	0.06	0.11	0.05
Weight adjusted mean aIMT (mm/kg)	0.033 (0.030, 0.041)	0.037 (0.029, 0.044)	0.71	0.97	0.25
Mean aIMT/aortic lumen diameter	0.071 ± 0.01	0.075 ± 0.01	0.32	0.47	0.20
Weight adjusted max aIMT (mm/kg)	0.036 (0.033, 0.044)	0.041 (0.032, 0.049)	0.71	0.89	0.23
Max aIMT/aortic lumen diameter	0.077 (0.071, 0.090)	0.079 (0.075, 0.088)	0.33	0.43	0.14
**Arterial stiffness**	*N* = 24	*N* = 29			
PWV (m/s)	4.09 ± 0.51	4.18 ± 0.53	0.54	0.51	0.34
**Central blood pressure**	*N* = 26	*N* = 32			
Central systolic blood pressure (mmHg)	93.7 ±5.4	91.4 ± 7.1	0.17	0.18	**0.04**
Central diastolic blood pressure (mmHg)	67.0 ± 4.4	66.3 ± 6.9	0.67	0.60	0.15
Central pulse pressure (mmHg)	26.5 (24.3, 29.3)	25.2 (22.1, 27.6)	0.05	0.06	0.09

Data presented as mean ± SD or median (IQR).

a Adjusted for age and gender.

b Adjusted for BMI and birth weight.

aIMT, aortic intima-media thickness; PWV, carotid-femoral pulse wave velocity.

The bold values indicate the statistical significance (*p* < 0.05).

Maternal characteristics are described in Table 3. Mothers with preeclampsia had a higher BMI at dating scan (24.0 vs. 22.4), higher rates of cesarean section (58% vs. 5%), antihypertensive medication (60% vs. 0%) and exercised more at 6 months (2 vs. 1 days). Otherwise, characteristics of mothers were not different. Mothers with preeclampsia were classified as early vs. late onset (19% vs. 81%) and severe hypertension preeclampsia vs. non severe (58% vs. 42%).

**Table 3:** Maternal characteristics stratified by preeclampsia vs. normotensive pregnancy.

	**Preeclampsia *n* = 26**	**Control *n* = 34**	***p value***
Age at birth (years)	32.5 ± 4.8	32.6 ± 3.9	0.91
Smoking ever at 6 months	9 (35)	9 (27)	0.49
Alcoholic drinks/week at 6 months (drinks)	1 (0, 2)	0 (0, 2)	0.44
Moderate exercise/week at 6 months (days)	2 (1,3)	1 (0, 2.25)	0.05
Illicit drug use ever at 6 months	7 (27)	16 (47)	0.11
Antihypertensive medication during pregnancy	15 (60)	0 (0)	**<0.001**
Parity			0.36
1	19 (73)	21 (62)	
2	7 (27)	13 (38)	
Ethnicity: Oceanian	14 (54)	18 (53)	0.94
Pre-pregnancy BMI (kg/m^2^)^*^	24.0(22.6, 27.0)	22.4 (20.9, 24.0)	**0.02**
Dating scan weight (kg)	64 (58.8, 69.0)	58.5 (54.5, 66.3)	0.06
Cesarean section	15 (58)	5 (15)	**<0.001**
Education			0.07
Tertiary	8 (31)	4 (12)	
University	18 (69)	30 (88)	
GDM/GDM in previous pregnancy	4 (15)	2 (6)	0.22
Preeclampsia onset			
Early	5 (19)	–	–
Late	21 (81)	–	
Preeclampsia blood pressure severity			
Hypertension^a^	11 (42)	–	–
Severe hypertension^b^	15 (58)	–	

Data presented as n (%), mean ± SD or median (IQR).

Categorical data calculates with Chi square or Fisher's exact.

BMI, body mass index; DBP, diastolic blood pressure; GDM, gestational diabetes mellitus; SBP, systolic blood pressure.

* Based on self-reported weight.

a Defined as Systolic blood pressure ≥140 mmHg and/or diastolic blood pressure ≥90 mmHg.

b Defined as Systolic blood pressure ≥160 mmHg and/or diastolic blood pressure ≥110 mmHg.

The bold values indicate the statistical significance (*p* < 0.05).

There were no significant associations between vascular measurements and explanatory variables in univariate analysis. Moreover, there were also no differences in aIMT or PWV in sub-group analysis by preeclampsia onset, BP severity and gestational age (results not shown).

## Discussion

In this cohort of children exposed to preeclampsia and controls, there were no major adverse differences in vascular structure or function. This contrasts with our hypothesis but reassuringly suggests that exposure to preeclampsia does not lead to vascular structure and function aberrations consistent with signs of early CVD in young children.

In our study, PWV was not different between participants exposed to intrauterine preeclampsia compared with controls. To the best of our knowledge, only two other studies have measured PWV in children exposed to intrauterine preeclampsia. One study from Pakistan with children aged 2–10 years (mean age 5 ± 2.2 years) reported a significant, albeit small, difference compared with controls (0.42 vs. 0.39 m/s) (21). However, participants exposed to preeclampsia had a lower gestational age, lower birthweight and higher rates of preterm birth compared to children in our study. Similarly to our study, the Avon Longitudinal Study of Parents and Children found no difference in carotid-radial PWV between groups in children and early adolescents (22). Our recent systematic review of exposures during the first 1,000 days of life suggest that changes to PWV may not manifest until adolescence (32).

Two other studies (18, 19) have investigated the association between intrauterine preeclampsia exposure and aIMT in childhood. Both reported a greater difference in aIMT compared with controls which contrasts with our findings. However, these studies may not be representative globally. In the study from Greece by Oikonomou (19), aIMT was measured in neonates during the first 5 days of life. This study included only neonates exposed to early onset preeclampsia, which is often associated with fetal complications including growth restriction compared to late-onset (33) and may have influenced aIMT. In our study, preeclampsia was predominately late onset which is reflective of the overall population distribution of early vs. late-onset preeclampsia (34) and therefore our results may be more generalizable. The rate of gestational diabetes was also higher in Oikonomou's preeclampsia group compared to controls (31% vs. 8%) (19) and may have also influenced the findings as maternal diabetes is known to influence aIMT (16, 35).

The presence of other CVD risk factors may explain differences between our results and previous studies. Neonates in a cohort from Turkey exposed to intrauterine preeclampsia had significantly higher triglycerides (2.2 vs. 0.3 mmol/L) and lower high density lipoprotein (1.0 vs. 1.5 mmol/L) compared to controls (18). Triglycerides were also significantly higher in mothers of exposed neonates compared to controls (12.4 vs. 7.2 mmol/L). Although lipids are elevated in women with preeclampsia compared to controls (36, 37), chronic and transient maternal hypercholesterolaemia are associated with increased fatty streak formation in the fetal aorta compared to the aortas of fetuses from mothers with normal cholesterol (38). In neonates, evidence of elevation of lipids following preeclampsia exposure is conflicting. A systematic review of the effect of preeclampsia exposure on the lipid concentration of cord blood found that three of six studies reported a significant increase in triglycerides compared to controls (3). Additional studies have reported a significant increase in lipids in children exposed to preeclampsia compared with controls (39) and another found no differences between groups (40) however, it is not known how long triglycerides persist as longitudinal studies have not been performed. As elevated triglyceride levels are associated with greater aIMT of neonates exposed to other adverse pregnancy complications such as growth restriction (35) or born large for gestational age (16), we speculate that elevated neonate lipids levels may have influenced the aIMT in the study by Akcakus et al. (18). Meta-analysis have reported that LDL is greater and HDL is lower in the cord blood of offspring exposed to intrauterine preeclampsia, however there were no differences in the lipids of children, adolescents and young children exposed to preeclampsia (3). While we did not assess lipids in this study, they should be investigated in future research.

There are challenges in assessing PWV and aIMT in young children. While feasible (41), we had to exclude five participants as they were unable to cooperate or keep still during the vascular assessments. Moreover, distractions were required in 40% of participants in order to sufficiently perform the assessments. A further four participants were excluded as they did not meet quality control. Despite this, we obtained more successful measurements than other studies which measured aIMT (41) or PWV (41, 42) in early childhood populations.

The effects of exposure to intrauterine preeclampsia may vary throughout childhood. As our study was conducted in children aged two to five, we cannot rule out the possibility of a regression in intimal thickening after birth. This is supported by a series of post mortem studies by Stary (43), where the frequency of fatty streaks located in the coronary arteries decreased after infancy and during early childhood before increasing in late childhood. This suggests that early lesions found in infancy were formed *in-utero* and are reflective of maternal risk factors. Accordingly, vascular health markers assessed in childhood may be more indicative of exposure to childhood risk factors. Longitudinal studies should seek to establish whether vascular health changes seen in newborn offspring exposed to preeclampsia regress during infancy. On the other hand, the time point of our study may be too early to detect clinically significant changes. A systematic review of 53,029 individuals investigated the association between exposure to intrauterine preeclampsia with cardiovascular risk factors and reported elevated BP and a mild increase in BMI from childhood and in early adulthood (3) however, there was considerable heterogeneity. We also found a mild increase in central systolic BP in children exposed to preeclampsia, after adjusting for BMI and birth weight. This is an important finding because elevated BP tracks from childhood to adulthood and is likely to predict hypertension in adulthood (44), highlighting the potential for early intervention in high-risk children. Although limited, evidence from the aforementioned systematic review (3) also indicated that vascular function is altered, with changes to endothelial function and cardiac morphometry reported in children exposed to preeclampsia compared to controls. PWV or IMT were not reported in this systematic review.

Although preeclampsia exposure was not associated with adverse cardiovascular markers in our cohort, there is growing evidence of the long term effects of offspring exposed to intrauterine preeclampsia including increased cardiovascular morbidity, elevated BMI and BP (4). Therefore those exposed to intrauterine preeclampsia may benefit from routine screening and monitoring of cardiovascular risk factors, particularly BP, to identify individuals at the greatest risk and to target intervention strategies. Routine screening may also be important as preeclampsia exposure often overlaps with other adverse outcomes in pregnancy such as low birth weight and preterm birth, which are both independently associated with increased CVD risk in later life (45, 46). However, the results of our study provide reassurance to parents of young children exposed to in intrauterine preeclampsia, as signs of increased CVD risk were not present in this cohort of children aged 2–5 years.

Strengths of the study include recruitment of participants in this sub-study from an established prospective cohort study of ethnically diverse women and their children as well as a large parallel control group (23). The participants are well characterized from a cohort that is broadly reflective of the overall preeclampsia population i.e., mostly late onset and without severe fetal growth restriction, and were assessed over a narrow age range. However, future studies which include a mix of exposure to early and late onset may be helpful in determining the impact of intrauterine preeclampsia exposure on vascular health. We followed best practice procedures for data collection (6, 47), using validated and acceptable methods for assessing early arterial injury in children (42, 48, 49), which contrasts other published studies (20, 32). Other adverse complications such as low birth weight and preterm birth increase CVD risk in later life (45, 46), however, we found no associations between aIMT or PWV and confounders in univariate or sub-group analysis. Due to slow recruitment and smaller than expected enrolment in to the study, we were unable to match cases vs. controls as initially planned. Whilst we recognize the study was potentially underpowered, none of the findings approached statistical or clinical significance. Further research, including longitudinal follow-up, is required to determine if alterations to vascular structure and function are apparent later in life.

We found no differences in vascular structure or function in 2–5 year old children as a result of intrauterine preeclampsia exposure. While this finding is reassuring, more research is required in larger cohorts with longitudinal follow up to determine if, and when, exposure to intrauterine preeclampsia affects the vascular health of children.

## Data availability statement

Data from this study will not be made available because accessing patient level data requires an application and permissions. Requests to access the datasets should be directed to megan.gow@health.nsw.gov.au.

## Ethics statement

The studies involving human participants were reviewed and approved by South Eastern Sydney Local Health District Human Research Ethics Committee (2019/ETH11984). Written informed consent to participate in this study was provided by the participants' legal guardian/next of kin.

## Author contributions

BV collected the data, carried out data analysis, interpreted the results, drafted the initial draft of the manuscript, and revised the manuscript. AH and GD conceived the study and design (P4 study) and critically reviewed the manuscript. LR collected the data (P4 study) and critically reviewed the manuscript. MS critically reviewed the manuscript and contributed to the study design. MC and MG conceived the study, were involved in interpretation of results and critically reviewed the manuscript. All authors approved the final manuscript as submitted and agree to be accountable for all aspects of the work.

## References

[1] Chappell LC Cluver CA Kingdom J Tong S. Pre-eclampsia. Lancet. (2021) 398:341–54. 10.1016/S0140-6736(20)32335-7 34051884

[2] Mol BWJ Roberts CT Thangaratinam S Magee LA de Groot CJM Hofmeyr GJ. Pre-eclampsia. Lancet. (2016) 387:999–1011. 10.1016/S0140-6736(15)00070-7 26342729

[3] Andraweera PH Lassi ZS. Cardiovascular risk factors in offspring of preeclamptic pregnancies—systematic review and meta-analysis. J Pediatr. (2019) 208:104–13.e6. 10.1016/j.jpeds.2018.12.008 30876753

[4] Wojczakowski W Kimber-Trojnar Z Dziwisz F Słodzińska M Słodziński H Leszczyńska-Gorzelak B. Preeclampsia and cardiovascular risk for offspring. J Clin Med. (2021) 10:3154. 10.3390/jcm10143154 34300320 PMC8306208

[5] Urbina EM Williams RV Alpert BS Collins RT Daniels SR Hayman L. Noninvasive assessment of subclinical atherosclerosis in children and adolescents. Hypertension. (2009) 54:919–50. 10.1161/HYPERTENSIONAHA.109.192639 19729599

[6] Skilton RM Celermajer SD Cosmi E Crispi F Gidding SS Raitakari TO. Natural history of atherosclerosis and abdominal aortic intima-media thickness: rationale, evidence, and best practice for detection of atherosclerosis in the young. J Clin Med. (2019) 8:1201. 10.3390/jcm8081201 31408952 PMC6723244

[7] Dalla Pozza R Ehringer-Schetitska D Fritsch P Jokinen E Petropoulos A Oberhoffer R. Intima media thickness measurement in children: a statement from the Association for European Paediatric Cardiology (AEPC) Working Group on Cardiovascular Prevention Endorsed by the Association for European Paediatric Cardiology. Atherosclerosis. (2015) 238:380–7. 10.1016/j.atherosclerosis.2014.12.029 25555270

[8] van den Oord SCH Sijbrands EJG ten Kate GL van Klaveren D van Domburg RT van der Steen AFW. Carotid intima-media thickness for cardiovascular risk assessment: systematic review and meta-analysis. Atherosclerosis. (2013) 228:1–11. 10.1016/j.atherosclerosis.2013.01.025 23395523

[9] Lorenz MW Markus HS Bots ML Rosvall M Sitzer M. Prediction of clinical cardiovascular events with carotid intima-media thickness. Circulation. (2007) 115:459–67. 10.1161/CIRCULATIONAHA.106.628875 17242284

[10] Ben-Shlomo Y Spears M Boustred C May M Anderson SG Benjamin EJ. Aortic pulse wave velocity improves cardiovascular event prediction: an individual participant meta-analysis of prospective observational data from 17,635 subjects. J Am Coll Cardiol. (2014) 63:636–46. 10.1016/j.jacc.2013.09.063 24239664 PMC4401072

[11] Epure AM Rios-Leyvraz M Anker D Di Bernardo S da Costa BR Chiolero A. Risk factors during first 1,000 days of life for carotid intima-media thickness in infants, children, and adolescents: a systematic review with meta-analyses. PLoS Med. (2020) 17:e1003414. 10.1371/journal.pmed.1003414 33226997 PMC7682901

[12] Dawson JD Sonka M Blecha MB Lin W Davis PH. Risk factors associated with aortic and carotid intima-media thickness in adolescents and young adults: the muscatine offspring study. J Am Coll Cardiol. (2009) 53:2273–9. 10.1016/j.jacc.2009.03.026 19520251 PMC2747309

[13] Järvisalo MJ Jartti L Näntö-Salonen K Irjala K Rönnemaa T Hartiala Jaakko J. Increased aortic intima-media thickness: a marker of preclinical atherosclerosis in high-risk children. Circulation. (2001) 104:2943–7. 10.1161/hc4901.100522 11739310

[14] Gomez-Roig MD Mazarico E Valladares E Guirado L Fernandez-Arias M Vela A. Aortic intima-media thickness and aortic diameter in small for gestational age and growth restricted fetuses. PLoS ONE. (2015) 10:e0126842. 10.1371/journal.pone.0126842 26017141 PMC4446260

[15] Akazawa Y Hachiya A Yamazaki S Kawasaki Y Nakamura C Takeuchi Y. Cardiovascular remodeling and dysfunction across a range of growth restriction severity in small for gestational age infants—implications for fetal programming. Circ J. (2016) 80:2212–20. 10.1253/circj.CJ-16-0352 27535477

[16] Akcakus M Koklu E Baykan A Yikilmaz A Coskun A Gunes T. Macrosomic newborns of diabetic mothers are associated with increased aortic intima-media thickness and lipid concentrations. Horm Res Paediatr. (2007) 67:277–83. 10.1159/000098157 17191031

[17] Koklu E Kurtoglu S Akcakus M Yikilmaz A Gunes T. Serum insulin-like growth factor-I (Igf-I) Igf binding protein-3 (Igfbp-3) and leptin levels are related to abdominal aortic intima-media thickness in macrosomic newborns. Growth Horm IGF Res. (2007) 17:26–32. 10.1016/j.ghir.2006.10.002 17113804

[18] Akcakus M Altunay L Yikilmaz A Yazici C Koklu E. The relationship between abdominal aortic intima-media thickness and lipid profile in neonates born to mothers with preeclampsia. J Pediatr Endocrinol Metab. (2010) 23:1143. 10.1515/jpem.2010.179 21284327

[19] Oikonomou N Fouzas S Gkentzi D Dimitriou G Karatza AA. Aortic intima-media thickness in neonates exposed to early-onset preeclampsia. Early Hum Dev. (2020) 151:105166. 10.1016/j.earlhumdev.2020.105166 32889166

[20] Varley BJ Nasir RF Skilton MR Craig ME Gow ML. Early life determinants of vascular structure in fetuses, infants, children, and adolescents: a systematic review and meta-analysis. J Pediatr. (2022) 2022:1–10. 10.1016/j.jpeds.2022.08.033 36029824

[21] Hoodbhoy Z Aslam N Mohsin S Ashiqali S Ali H Sattar S. Cardiovascular dysfunction in children exposed to preeclampsia during fetal life. J Am Soc Echocardiogr. (2021). 10.1016/j.echo.2021.01.008 33453366

[22] Lawlor DA Macdonald-Wallis C Fraser A Nelson SM Hingorani A Davey Smith G. Cardiovascular biomarkers and vascular function during childhood in the offspring of mothers with hypertensive disorders of pregnancy: findings from the avon longitudinal study of parents and children. Eur Heart J. (2012) 33:335–45. 10.1093/eurheartj/ehr300 21862461 PMC3270043

[23] Davis GK Roberts L Mangos G Henry A Pettit F O'Sullivan A. Postpartum physiology, psychology and paediatric follow up study (p4 study)—study protocol. Pregnancy Hypertens. (2016) 6:374–9. 10.1016/j.preghy.2016.08.241 27939485

[24] Brown MA Magee LA Kenny LC Karumanchi SA McCarthy FP Saito S. The hypertensive disorders of pregnancy: ISSHP classification, diagnosis and management recommendations for international practice. Pregnancy Hypertens. (2018) 13:291–310. 10.1016/j.preghy.2018.05.004 29803330

[25] Magee LA Brown MA Hall DR Gupte S Hennessy A Karumanchi SA. The 2021 international society for the study of hypertension in pregnancy classification, diagnosis and management recommendations for international practice. Pregnancy Hypertens. (2022) 27:148–69. 10.1016/j.preghy.2021.09.008 35066406

[26] Tranquilli AL Brown MA Zeeman GG Dekker G Sibai BM. The definition of severe and early-onset preeclampsia. Statements from the International Society for the Study of Hypertension in Pregnancy (ISSHP). Pregnancy Hypertens. (2013) 3:44–7. 10.1016/j.preghy.2012.11.001 26105740

[27] Skilton MR Evans N Griffiths KA Harmer JA Celermajer DS. Aortic Wall Thickness in newborns with intrauterine growth restriction. Lancet. (2005) 365:1484–6. 10.1016/S0140-6736(05)66419-7 15850633

[28] World Health Organization. Child Growth Standards. (2022). Available online at: https://www.who.int/tools/child-growth-standards/software (accessed March 30, 2022).

[29] Villar J Giuliani F Bhutta ZA Bertino E Ohuma EO Ismail LC. Postnatal growth standards for preterm infants: the preterm postnatal follow-up study of the intergrowth-21st project. Lancet Glob Health. (2015) 3:e681–e91. 10.1016/S2214-109X(15)00163-1 26475015

[30] Cai TY Meroni A Dissanayake H Phang M Avolio A Celermajer DS. Validation of a cuff-based device for measuring carotid-femoral pulse wave velocity in children and adolescents. J Hum Hypertens. (2019) 34:311–8. 10.1038/s41371-019-0191-1 30877274

[31] AtCorMedical Pty Ltd. Operator's Manual Sphygmocor Xcel System V1. Sydney, NSW: AtCor Medical Pty Ltd (2020).

[32] Varley BJ Nasir RF Craig ME Gow ML. Early life determinants of arterial stiffness in neonates, infants, children and adolescents: a systematic review and meta-analysis. Atherosclerosis. (2022) 355:1–7. 10.1016/j.atherosclerosis.2022.07.006 35841718

[33] Raymond D Peterson E. A critical review of early-onset and late-onset preeclampsia. Obstet Gynecol Surv. (2011) 66:97–506. 10.1097/OGX.0b013e3182331028 22018452

[34] Lisonkova S Joseph KS. Incidence of preeclampsia: risk factors and outcomes associated with early- versus late-onset disease. Am J Obstet Gynecol. (2013) 209:544.e1–e12. 10.1016/j.ajog.2013.08.019 23973398

[35] Koklu E Kurtoglu S Akcakus M Koklu S Buyukkayhan D Gumus H. Increased aortic intima-media thickness is related to lipid profile in newborns with intrauterine growth restriction. Horm Res. (2006) 65:269–75. 10.1159/000092536 16601348

[36] Gallos ID Sivakumar K Kilby MD Coomarasamy A Thangaratinam S Vatish M. Pre-eclampsia is associated with, and preceded by, hypertriglyceridaemia: a meta-analysis. BJOG. (2013) 120:1321–32. 10.1111/1471-0528.12375 23859707

[37] Spracklen CN Smith CJ Saftlas AF Robinson JG Ryckman KK. Maternal hyperlipidemia and the risk of preeclampsia: a meta-analysis. Am J Epidemiol. (2014) 180:346–58. 10.1093/aje/kwu145 24989239 PMC4565654

[38] Napoli C D'Armiento FP Mancini FP Postiglione A Witztum JL Palumbo G. Fatty streak formation occurs in human fetal aortas and is greatly enhanced by maternal hypercholesterolemia. Intimal accumulation of low density lipoprotein and its oxidation precede monocyte recruitment into early atherosclerotic lesions. J Clin Invest. (1997) 100:2680–90. 10.1172/JCI119813 9389731 PMC508471

[39] Kharb S Nanda S. Patterns of biomarkers in cord blood during pregnancy and preeclampsia. Curr Hypertens Rev. (2017) 13:57–64. 10.2174/1573402113666170126101914 28128050

[40] Alahakoon TI Medbury HJ Williams H Lee VW. Lipid profiling in maternal and fetal circulations in preeclampsia and fetal growth restriction-a prospective case control observational study. BMC Preg Childbirth. (2020) 20:61. 10.1186/s12884-020-2753-1 32000699 PMC6993402

[41] Zhao B Johnston FH Dalton M Negishi K. Feasibility and normal ranges of arterial intima-media thickness and stiffness in 2-year-old children: a pilot study. Pediatr Cardiol. (2019) 40:914–20. 10.1007/s00246-019-02088-1 30879086

[42] Keehn L Milne L McNeill K Chowienczyk P Sinha MD. Measurement of pulse wave velocity in children: comparison of volumetric and tonometric sensors, brachial-femoral and carotid-femoral pathways. J Hypertens. (2014) 32:1464–9. 10.1097/HJH.0000000000000203 24759123 PMC4059550

[43] Stary HC. Lipid and macrophage accumulations in arteries of children and the development of atherosclerosis. Am J Clin Nutr. (2000) 72:1297s−306s. 10.1093/ajcn/72.5.1297s 11063472

[44] Chen X Wang Y. Tracking of blood pressure from childhood to adulthood. Circulation. (2008) 117:3171–80. 10.1161/CIRCULATIONAHA.107.730366 18559702 PMC3568631

[45] Belbasis L Savvidou MD Kanu C Evangelou E Tzoulaki I. Birth weight in relation to health and disease in later life: an umbrella review of systematic reviews and meta-analyses. BMC Med. (2016) 14:147. 10.1186/s12916-016-0692-5 27677312 PMC5039803

[46] Markopoulou P Papanikolaou E Analytis A Zoumakis E Siahanidou T. Preterm birth as a risk factor for metabolic syndrome and cardiovascular disease in adult life: a systematic review and meta-analysis. J Pediatr. (2019) 210:69–80.e5. 10.1016/j.jpeds.2019.02.041 30992219

[47] Van Bortel LM Laurent S Boutouyrie P Chowienczyk P Cruickshank JK De Backer T. Expert consensus document on the measurement of aortic stiffness in daily practice using carotid-femoral pulse wave velocity. J Hypertens. (2012) 30:445–8. 10.1097/HJH.0b013e32834fa8b0 22278144

[48] Stabouli S Printza N Zervas C Dotis J Chrysaidou K Maliahova O. Comparison of the sphygmocor xcel device with applanation tonometry for pulse wave velocity and central blood pressure assessment in youth. J Hypertens. (2019) 37:30–6. 10.1097/HJH.0000000000001819 29939943

[49] McCloskey K Ponsonby A-L Carlin JB Jachno K Cheung M Skilton MR. Reproducibility of aortic intima-media thickness in infants using edge-detection software and manual caliper measurements. Cardiovasc Ultrasound. (2014) 12:18. 10.1186/1476-7120-12-18 24894574 PMC4061507

